# Exploring the barriers to patient engagement in the delivery of safe care in Iranian hospitals: A qualitative study

**DOI:** 10.1002/nop2.411

**Published:** 2019-11-25

**Authors:** Zahra Chegini, Ali Janati, Javad Babaie, Mahboub Pouraghaei

**Affiliations:** ^1^ Department of Health Services Management Faculty of Management and Medical Informatics Tabriz University of Medical Sciences Tabriz Iran; ^2^ Iranian Center of Excellence in Health Management School of Management and Medical Informatics Tabriz University of Medical Sciences Tabriz Iran; ^3^ Emergency Medicine Research Team Faculty of Medicine Tabriz University of Medical Sciences Tabriz Iran

**Keywords:** expert opinion, Iran, nurses, patient engagement, patient involvement, patient participation, patient safety, qualitative research

## Abstract

**Aim:**

To investigate barriers to patient engagement in the delivery of safe hospital care.

**Design:**

Qualitative exploratory study.

**Methods:**

A qualitative study with 35 Iranian health professionals was conducted from February to April 2019 using semi‐structured interviews to elicit their opinions. MAXQDA 11 software was used for data management, and the data were analysed using framework analysis.

**Results:**

Barriers, which potentially have negative impact on patient engagement in the delivery of safer care, were categorized into four themes. The first category included patient‐related barriers such as low levels of health literacy, ineffective education, patient unwillingness and cultural barriers. The second category included staff‐related barriers such as the existence of negative attitudes towards engaging patients in matters relating to patient safety, ineffective communication, high workload and the reluctance on the part of physicians to engage with patients. Barriers created by limited resources and inadequate training provided by universities and in the workplace formed the third category and community‐related barriers such as the inadequate dissemination of information via the mass media and a lack of community‐based services formed the fourth category.

**Conclusion:**

Results demonstrate the multilayered nature of the significant barriers to the engagement of patients in the delivery of safe care and reflect the need for a collaborative approach between the recipients of care, researchers, care providers and policy makers if these are to be overcome.

## 1 INTRODUCTION

International research has shown that significant numbers of harmful incidents, many of which are preventable, occur in hospitals (Donaldson, Corrigan, & Kohn, 2000). It is in response to this research that patient safety has emerged as an area of interest and per se has turned into the subject of much healthcare research. Achieving patient safety guarantees patients' freedom from accidental or preventable injuries whilst under medical care and, as such, might be considered a right or an entitlement that patients have (Yu, Flott, Chainani, Fontana, & Darzi, 2016). Research has shown that, on average, patients are harmed in 10% of all hospital admissions, and it is estimated that up to 75% of these incidents are preventable (WHO, 2013a).

A core theme of the World Health Organization's (WHO) programme, "Patients for Patient Safety" is that engaging patients at all levels in the health system is essential if their ability to improve patient safety and the quality of care is to be enhanced. Improved treatment outcomes and increased patient trust and satisfaction have been shown to be associated with patient engagement (Vahdat, Hamzehgardeshi, Hessam, & Hamzehgardeshi, 2014) and, with findings such as these, patients should undoubtedly be at the centre of all healthcare activities.

## 2 BACKGROUND

In health care, the term “patient engagement” has been used to describe a situation where patients have been involved in matters such as decision‐making, self‐medication, self‐monitoring, patient education, goal setting and taking part in physical care (Cahill, 1998). However, whilst the concept of “patient engagement” is recognized, unanimous definition of patient engagement exists, rather a variety of terms, including “patient involvement,” “patient collaboration,” “patient empowerment,” “partnership” and “patient‐centered care,” have been used to describe a partnership with patients (Cahill, 1998;Longtin et al., 2010). This study adopted a Patient Engagement Framework developed in an earlier study (Kim et al., 2018). This framework is based on Arnstein's ladder of citizen participation (Arnstein, 1969), which describes a spectrum ranging from non‐participation to citizen control.

To involve patients in matters of patient safety, it requires patients to be made aware of safety issues without creating a sense of fear in them. It is evident, then, that to involve patients in matters of patient safety requires a difficult balancing act and examples of how this has been done successfully include the Clean Your Hands and Speak Up campaigns conducted in the UK and the USA, respectively (Pinto, 2007).

In order to accentuate the significance of safety issues in hospitals, the WHO Eastern Mediterranean Regional Office (EMRO) developed the patient safety friendly hospital initiative (PSFHI) in 2007. At first, six countries were chosen as candidate to perform the programme and later it was implemented in all other countries in the region (Bairami, Ghorbanpoor, & Bairami, 2015). Iran, as one of the countries of the region, participated in the programme. In the first step, 10 hospitals from around the country were selected as pilot phase. Then and according to achievements, the Ministry of Health and Medical Education (MoHME) ordered it to be implemented in about 100 hospitals in the country.

However, an evaluation of the PSFHI programme in seven countries covered by the WHO EMRO indicated that none of the participating hospitals met the minimum compulsory standards required for the hospitals enrolled in the program (Siddiqi et al., 2012). Studies evaluating the status of patient safety friendly standards in Iranian hospitals show poor status of standards for attracting patients and community participation (Asefzade, Mehrabian, Nikpey, & Kianmehr, 2013;Mazhari & Adel, 2015) and has also shown that patient engagement is afforded little respect in the Iranian healthcare system (Atoof et al., 2015). Another qualitative study (Hooshmand, Tourani, Ravaghi, & Ebrahimipour, 2014) suggests encouraging patients' participation in the treatment process, advocating reorganization culture and concentrating on patient safety, patients' satisfaction and effective dealing with complaints to make the health system more patient‐oriented.

A review of existing literature reveals that whilst studies examining patient engagement in patient safety have been conducted in developed countries (Martin, Navne, & Lipczak, 2013;Ringdal, Chaboyer, Ulin, Bucknall, & Oxelmark, 2017;Skagerstrom, Ericsson, Nilsen, Ekstedt, & Schildmeijer, 2017), and there is a lack of evidence from developing countries. In developing countries, improvements in levels of patient safety require a holistic approach that has a clear vision emanating from the political leadership and which, above all, puts patients at the centre of the care process (Elmontsri, Banarsee, & Majeed, 2018).

Whilst research shows that patients are willing and capable of participating in patient safety initiatives (Davis, Sevdalis, & Vincent, 2011;Entwistle et al., 2010;Zhang et al., 2012), there remains an ambiguity over how they can become engaged in patient safety activities (Armstrong, Herbert, Aveling, Dixon‐Woods, & Martin, 2013;Hall et al., 2010), and whilst evidence of patient engagement in other aspects of health care has been well‐documented, as regards patient safety, engagement remains an emerging field of interest with limited evidence (Chegini, Arab‐Zozani, & Janati, 2019;Davis, Jacklin, Sevdalis, & Vincent, 2007).

Having an understanding of those factors, which can act as a barrier undermining the engagement of patients in practices that address safety, can help to reduce the number of adverse events (Scobie & Persaud, 2010). Therefore, the objective of this study was to obtain qualitative information from healthcare professionals (HCPs) that describe their experiences of patient engagement and their attitudes towards it.

## 3 METHODS

### 3.1 Aims

To investigate attitudes of Iranian HCPs regarding the barriers to patient engagement in the delivery of safe hospital care.

### 3.2 Design

This was a qualitative study based on semi‐structured interviews and was conducted in Iran during February–April 2019. A more detailed outline of the methods that were adopted and the research questions that were posed may be found in the study protocol document (Chegini, Janati, Babaie, & Pouraghaei, 2019).

### 3.3 Sampling and recruitment

For selecting key informants, maximum variation sampling technique was used, which allowed selecting the participants who exhibited a wide range of experiences, which was significant in gaining greater insights (Green & Thorogood, 2018). Participants were selected based on the following inclusion criteria:

1. Members of hospital staff with responsibility, accountability and authority for patient safety and whose work actively involve the coordination of patient safety in a hospital.
2. Individuals at the Ministry of Health (MOH) responsible for the accreditation of patient safety standards. Also, included in this category were staff from the department of quality and patient safety and from the department of the Vice Chancellor for Treatment Affairs at Medical Universities.
3. Academics with a background and experience in patient safety.

The Research Vice‐Chancellor of Tabriz University of Medical Science sent a formal letter explaining the study to MOH and the Medical Universities of three provinces (Tehran, Tabriz and Qazvin) explaining the objectives of the study and introducing the investigators. The selection process was at first purposeful (expert selection) and was completed in snowball manner (Patton, 1990). To put it differently, the sampling process was started with a convenience sample of a few information‐rich cases of participants based on their availability and willingness. They were told to recommend others for researchers to contact. These, in turn, are asked to suggest more participants.

### 3.4 Data collection

Data collection was by way of semi‐structured interviews. Participants were initially contacted by one of the researchers (ZCh) by telephone or via email to agree a mutually convenient time for an interview to be conducted with two of the researchers (ZCh and AJ). Subsequently, 35 interviews took place. Analysis proceeded simultaneously with data collection by comparing data from one interview with that from another. Interviewing ceased at the point that data saturation occurred, that is, when no more codes or themes were identified from the last two interviews. Interview duration ranged from 20–55 min and, with the consent of the participants, all interviews were audio‐recorded, transcribed and subsequently analysed.

### 3.5 Ethical considerations

Research Ethics Committee approval was obtained from the Tabriz University of Medical Science (ethical code: IR.TBZMED.REC.1397.617) on the basis that all the interviews would be tape‐recorded and transcribed and that participants had the right to withdraw from the study at any time without prejudice. Assurances were also provided by the researchers that the information collected during the interviews would be treated confidentially and that the anonymity of the participants would be protected.

### 3.6 Data analysis

Framework analysis was used as the method for data analysis. This was an iterative analytical five stage process involving the following: familiarization, identifying a thematic framework, indexing, charting and, finally, mapping and interpretation (Ritchie & Spencer, 2002) with MAXQDA 11 (VERBI GmbH) used at each stage. At the familiarization stage, a form, containing information about each participant and a summary of interview content, was prepared. An initial conceptual guideline was created by consensus between the researchers, and this was used as the basis for the assessment of each interview.

Each interview was separately coded by one of the researchers (ZCh) and a list of these codes was compiled and an association with the conceptual framework was extracted. At this stage, one or two specific codes were given to each section containing common interview information. In group discussion meetings with the other researchers, these codes were scrutinized and altered, if necessary. Then, at the charting stage, a comparison was drawn between the opinions expressed by the participants about each criterion and, if necessary, for a better understanding of what had been said during the interviews, the original interview was referred to and a note was added.

### 3.7 Rigour

To ensure the accuracy and validity of the study, the credibility, dependability and conformability of the data were evaluated. To address issues regarding validity, the participants were asked to share their opinions about the research findings and at each stage of the data analysis process the research team ensured that proper consideration was given to the issues raised. Documents generated at each stage of the research process were maintained so as to guarantee conformability. The COREQ guidelines were used throughout the study to enhance methodological rigour and trustworthiness (Tong, Sainsbury, & Craig, 2007).

## 4 FINDINGS

Thirty‐four face to face interviews took place, and one interview was conducted using Skype. The interviews involved experts from the MOH (*N* = 6), members of staff from the departments of the Vice Chancellor for Treatment Affairs at the Medical Universities (*N* = 5), hospital staff with responsibility for patient safety (*N* = 15) and experts with relevant qualifications and with at least one published paper on the subject (*N* = 9). Table 1 showed that most respondents were males (57.14%), with average age of 43.27 (*SD * = 7.34) years and average work experience of 10.15 (*SD* = 7.58). Most of the respondents had a degree of PhD (42.86%). This qualitative study identified 13 subthemes and four main themes. The percentage of any response related to a given theme was obtained by means of counting the number of times that theme was mentioned in the interviews (Table 2). The participants' illustrative statements during interview have been provided in online Supplementary Table.

**Table 1:** Descriptive characteristics of the participants

Demographics (*n* = 35)			
Qualitative variables		Frequency	%
Gender	Male	20	57.14
	Female	15	42.86
Professional position	Experts from the Ministry of Health (MOH)	6	17.14
	Members of staff from the Vice Chancellor for Treatment Affairs at the Medical Universities	5	14.29
	Patient safety experts at hospitals	15	42.86
	Faculty members and academics	9	25.71
Highest level of education	Bachelor	6	17.14
	Masters	10	28.57
	Ph.D.	15	42.86
	MD, Specialists	4	11.43

Quantitative variables	Minimum	Maximum	Mean	*SD*
Average age (years)	33	56	43.27	7.34
Average work experience (years)	1	32	10.15	7.58

**Table 2:** Framework analysis of the semi‐structured interviews

Themes	Subthemes	*N* (%)^a^
Patient‐related barriers	Low levels of health literacy	12 (34)
	Ineffective patient education	18 (51)
	Patient unwillingness	8 (23)
	Cultural barriers	15 (43)
Staff‐related barriers	Negative attitudes towards patient engagement	13 (37)
	Lack of effective communication	19 (54)
	Workload	11 (31)
	Reluctance of physicians	7 (20)
System‐related barriers	Limited resources	14 (40)
	The inadequate curriculum for health professionals	6 (17)
	Ineffective retraining programs	8 (23)
Community‐related barriers	Poor disseminate information via the mass media	15 (43)
	Lack of community‐based services	11 (31)

a Percentages are calculated out of a total of 35 participants who responded to the interview.

### 4.1 Patient‐related barriers

The study confirms that low level of health literacy created an obstacle to patient engagement in addressing patient safety. On the other hand, some expressed the view that low level of health literacy has a negative impact on the willingness of physicians to encourage patient engagement.

A further patient‐related barrier identified in the study concerned the perception amongst the participants that patients received little effective education about health and safe care issues, raising matters such as the failure to use simple and understandable language with patients and the absence of any assessment of what the patient has learned about their diagnosis and treatment. Moreover, the participants expressed concerns about the lack of continuous education available to patients (from the time the patient admission to hospital until their discharge) and that what education there was not flexible, and therefore, not capable of meeting the needs of different patients. For one participant, one of the biggest challenges is the need to address patients' pseudo education, especially on discharge.

Most participants expressed the view that the absence of a clear picture about engagement creates a barrier. The participants felt that staff did not completely explain the impact of patient participation in safety activities to patients and that it was this failure that meant the trust and willingness of patients to engage was not captured.

Finally, culture was cited as one of the patient‐related barriers. The participants offered the observation that patients come from different regions with different cultures and that these differences has an impact on the acceptance of treatment and on patient engagement. A culture of providing comprehensive medical information does not exist amongst Iranian patients yet.

### 4.2 Staff‐related barriers

The second theme, staff‐related barriers, consisted of four subthemes. Firstly, negative attitudes held by staff was cited as one of the barriers, which may pose difficulties, with the potential for an informed patient to meddle in staff duties being given as a reason for such negative attitudes.

Secondly, a lack of effective communication was identified as a barrier. Most participants expressed the view that staff do not communicate effectively with patients. Poor relationship between staff within teams was also noted by the participants.

Thirdly, staff‐related barrier identified was that of staff' workload and the limited time available to staff. However, one participant did suggest that workload cannot be an acceptable excuse and that nurses use it as a means to cover up their own failures.

Finally, a reluctance on the part of physicians was identified. In Iran, a power imbalance exists between physicians and other staff with physicians perceived at the higher level, holding significant authority yet most failing to follow patient safety requirements.

### 4.3 System‐related barriers

The third main theme, system‐related barriers, consisted of three subthemes. The first of these relates to resource limitation. The financial pressures experienced by hospitals mean that programs such as those engaging patients in safety are afforded the least priority by managers. According to one participant, even on patient safety walk‐rounds usually discussions turned to the equipment on wards and the facilities deficit rather than looking at safety issues.

A second system‐related barrier according to the participants is the poor curricula provided for health professionals. The participants felt that patient safety, patient engagement and teamwork were not emphasized enough at university. One participant believed that the curricula provided for managers also needed to be enriched.

A third subtheme identified was the ineffectiveness of retraining programs. It was felt that hospital managers can empower staff in these retraining courses or could clearly determine the domain of both patient and staff roles in the delivery of safe care.

### 4.4 Community‐related barriers

The fourth main theme, community‐related barriers, consisted of two subthemes. First, the poor dissemination of information via the mass media was mentioned. Most participants emphasized that whilst comprehensive health education programs using social media constitutes a strategy to improve patients health literacy, little effort has been made in this regard.

The final issue mentioned was the lack of community‐based services. The participants believed that charity groups or popular forums are absent in the Iranian health system despite the involvement of such groups having been recognized by the WHO as a means to involve patients in safety‐related matters. Community‐based organizations also have the potential to empower informal carers (family members who have responsibility for the care of unwell relatives) to practice safer care.

## 5 DISCUSSION

In this study, barriers to the engagement of patients in the delivery of safe care in Iran were identified. The present study showed that levels of health literacy are disproportionate to the number of patients and that this undermines patients' ability to take an active role in safety. A recent systematic review also highlighted the inadequacy of health literacy amongst the Iranian population, finding it to be borderline (Dadipoor, Ramezankhani, Aghamolaei, Rakhshani, & Safari‐Moradabadi, 2018). Having an adequate level of health literacy will assist the patient in a range of situations including being able to understand the problem, to use a variety of sources to obtain information and to make informed and shared decisions, such patients would be more likely to adhere to treatment (Ishikawa & Yano, 2008). Other studies have also suggested that the safety knowledge of both patients and professionals need to be improved if safer care is to be promoted (Sahlström, Partanen, Rathert, & Turunen, 2016;Schildmeijer, Nilsen, Ericsson, Broström, & Skagerström, 2018). This was also emphasized by Brabers, Rademakers, Groenewegen, Dijk, and Jong (2017) who argued that people with higher levels of health literacy are able to engage in a range of actions, which are aimed at enhancing their health.

Ineffective patient education and a failure in education at the time of discharge were another barrier identified in this study. These findings are in line with the results of Rainey, Ehrich, Mackintosh, and Sandall (2015) who found that a lack of knowledge about healthcare procedures and routines and how to detect and report changes in their clinical conditions meant that patients require both orientation and education. Schwappach (2010) has also suggested that healthcare professionals ought to see education of patients about safety issues challenging, but it is as a core element of their role and doing so will improve their own expertise. However, in a study examining the views of nurses, it was found that the workload of Iranian nurses, the limited time they had and the disproportionate number of patients in relation to the number of nursing staff all served to create barriers to patient education (Adib‐Hajbaghery & Zare, 2017). Discharge management practices such as those in the UK and in the Netherlands should be implemented, particularly as regards what signals or side‐effects they should look out for after their discharge from hospital (WHO, 2013a, 2013b).

This study also demonstrates that unwillingness on the part of patients is an important factor. This is in line with the findings from other research, which has shown that patients, worried whilst unwell, are not always prepared to commit the time and energy necessary to improve their care (Doherty & Stavropoulou, 2012). Similarly, there are difficulties associated with the physical and mental health of some patients (Schildmeijer et al., 2018) and with the unfamiliarity of patients with the healthcare environment and their limited understanding of their treatment and care (Bishop & Macdonald, 2017;Schwappach & Wernli, 2010). It was concluded by Tobiano, Bucknall, Sladdin, Whitty, and Chaboyer (2018) that, in medical wards, patients did have a desire for patient participation but their willingness to participate and the attitude of nurses can act to challenge active participation. In a study conducted by Waterman et al. (2006), 71% of patients were comfortable with the idea of helping the healthcare professional to mark the surgical site but only 17% actually did so. In this regard, it has been suggested by Davis et al. (2011) that encouragement from nurses may serve to increase patient willingness to participate in safety‐related behaviours.

Cultural barriers were also highlighted in this study. This reflects the findings of Schildmeijer et al. (2018) who found that the motivation of patients towards engagement was associated with their cultural background and also reflects the findings of a systematic review, which identified cultural factors including the hierarchical, paternalistic culture associated with the healthcare professions as presenting a barrier to the involvement of patients in safety matters (Doherty & Stavropoulou, 2012;Vaismoradi, Jordan, & Kangasniemi, 2015). Also, consistent with the present study are the results of a study, which found a significant relationship between various culture‐related variables and the degree of patient participation (Schouten, Meeuwesen, Tromp, & Harmsen, 2007). Health professionals should, therefore, consider the differences between their patients when exploring with them their “ideas, concerns and expectations” (Matthys et al., 2009).

Amongst the staff‐related barriers identified in this study, the negative attitude of staff was shown to be significant. If patients are to be engaged in healthcare, professional decisions must be more patient centred (Sahlström et al., 2016) and there needs to be a change in their attitude with a shift away from paternalism towards collaboration (Hubbard, Kidd, Donaghy, McDonald, & Kearney, 2007). This contrasts with the findings of a literature review regarding participation in decision‐making which found that generally staff had a positive attitude towards patient participation (Vahdat et al., 2014). Furthermore, in a study conducted in Sweden, it was found that a permissive attitude that allowed patients to ask questions, to seek advice and to state their opinion was important (Oxelmark, Ulin, Chaboyer, Bucknall, & Ringdal, 2018). Direct experience of participatory working is likely to lead to positive changes in the perceptions of professional staff (Forbat, Cayless, Knighting, Cornwell, & Kearney, 2009).

A lack of effective communication between HCPs and between HCPs and patients was another barrier identified in this study. This poor communication meant that patients were often ignored by staff, a finding which is in line with that of Ridelberg, Roback, and Nilsen (2014), a study conducted in Sweden. Ridelberg *et al* found that the interaction between patients and nurses was an important factor capable of hindering or facilitating patient safety depending on the quality of the communication between them. In another study, training patient communication was found to be an effective method of increasing the total level of active participation of patients in healthcare interactions. Improved communication could be achieved through an increased awareness amongst staff of how patient preferences for treatment can vary and by providing physicians with training in effective communication skills and in the use of visual representations, which are an effective but less commonly used approach (Schwartzberg, Cowett, VanGeest, & Wolf, 2007).

In this study, the participants believed that the workload of health professionals was the principle obstacle hindering effective patient‐provider communication and inhibiting patient engagement. The study conducted by Adib‐Hajbaghery and Zare (2017) also found workload in Iranian hospitals to be an important barrier hindering effective patient education. It was also found in a qualitative study conducted in 2017 that nursing participants often felt under pressure with a workload that sometimes meant that they could not adopt patient safety behaviours (Bishop & Macdonald, 2017). Hospitals desiring to create sufficient time for staff to engage in educating their patients might start by conducting a workflow analysis of hospital units (European Commission, 2012).

In contrast with the study conducted by Rashidian, Nedjat, Mounesan, Haghjou, and Majdzadeh (2015), the participants in this study believed that physicians are reluctant to engage with patients. In a Swedish study, physicians believed that pressure of time made it more difficult for them to maintain a focus on the patient, to give the patient their full attention, to achieve well‐functioning communication and to establish trusting relationships (Schildmeijer et al., 2018). As in many other countries, some Iranian physicians think that if they ask for the patient's opinion about a decision, they will be perceived as having a lack of knowledge and experience (Rahimi, Alizadeh, & Légaré, 2017). In Iran, patients are more typically interested in receiving information about their medical condition from their physician rather than being significantly involved in the decision‐making process (Mostafaie et al., 2014) and, as a result, most patients prefer their doctor to make the decisions (Mira, Guilabert, Pérez‐Jover, & Lorenzo, 2014). Therefore, physicians have an important role to play in engaging patients in safe care by exchanging information, building good interpersonal relationships and by sharing decision‐making (Sutker, 2008;Wu, 2000).

Resource limitation, another barrier identified by the participants in this study, was also highlighted in an earlier study (Rainey et al., 2015). Inadequate facilities, budget deficits and a lack of adequate medical equipment and medicines can contribute to stress and conflict for patients, their families and for staff (Nayeri & Negarandeh, 2009) leading to less effort being made to involve patients. Poor human resource planning and allocation and inadequate career pathways at all levels in the Iranian health system have all been identified as significant factors undermining the functional status of the health system (Tabrizi, Gholipour, Farahbakhsh, & Hasanzadeh, 2017).

According to the participants, education and training curricula for health professionals should pay greater attention to patient safety issues. A patient safety curriculum guide for medical schools was published by the WHO in 2009 and, in 2011, a multi‐professional version was published (WHO, 2009, 2011); however, the participants in this study felt these guides are rarely used in medical colleges. A review of existing literature suggests that some training programmes fail to demonstrate any worth in improving the quality of patient care (Fisher & Sadera, 2011). If capacities are to be expanded and educational outputs improved, training programs should enhance the potential and capabilities of employees (Naqvi & Khan, 2013).

The participants in this study felt that animated health information disseminated through national TV channels could promote knowledge of health‐related issues amongst the population. This follows the findings of other national studies examining knowledge improvement, which showed that using different forms of mass media can reach larger audiences (Aminian, Arbatani, Khajeheian, Zangi, & Shadmehr, 2013;Gholami, Pakdaman, Montazeri, & Virtanen, 2017 ). It has also been shown that mass media interventions in respect of health promotion have a more positive impact in developing countries than in developed countries (Tones & Tilford, 2001). Examples of how patients might be encouraged to become involved in safety matters include, from the US, the “Lehigh Valley Hospital and Health Network (LVHHN) Patient Safety Video” and, from the UK, the “Participate Inform Notice Know (PINK)” patient safety video (Pinto, Vincent, Darzi, & Davis, 2012).

The last barrier identified was the lack of community‐based services. In Iran, hospital‐based services are preferred over community‐based services (Heydari, Shahsavari, Hazini, & Nasrabadi, 2016), even though, in response to demographic change, community‐based care has been adopted across the world (Francesca, Ana, Jérôme, & Frits, 2011). Nationally, community organizations and civil society groups can influence policies and, at the local level, basing services in the community creates a new form of governance for the public health system and changes the relationship between providers and the users of health services (Wilson, Lavis, & Guta, 2012).

### 5.1 Strengths and limitations

One of the strengths of this study is the mixture of participants involved in the interviews. We included HCPs with a variety of expertise, which enlarged the variety of views. Whilst this study was conducted with rigour and its findings may be considered reliable, it does have some limitations. An important limitation relates to the immensity of the subject and the wide range of issues that are associated with patient engagement and, in this regard, it is recommended that each of the subjects identified in this study be explored in greater detail in a separate study. A further limitation is that the study only included participants from MOH and from three Iranian states (Tehran, Tabriz and Qazvin) and, as a result, transferability is limited. Any future studies should, therefore, include participants from other Iranian states. Lastly, it might be argued that this study is limited insofar as it only addressed the perspectives of healthcare professionals, however, the study forms part of a larger study exploring the views and perceptions that patients have regarding barriers to patient engagement in the delivery of safe care.

## 6 CONCLUSION

This study has the potential to raise awareness amongst health professionals and policy makers of the problems that Iranian hospitals face in engaging patients in taking measures to improve their safety and of how appropriate policies might be formulated. However, further research is needed to identify those actions necessary for patients to be engaged in patient safety issues and to define to what extent the active involvement of patients can be linked to improvements in patient safety and healthcare outcomes.

This study provides substantive evidence supporting the engagement of the patients in the delivery of safe care in hospitals particularly in developing countries and recognition of a need for strategies to overcome barriers to patient engagement. Such strategies need to focus on collaboration across multiple fields of expertise: in policy, in the community, in organizations and in interpersonal theories. Mechanisms that inform and empower patients are also required, as is improved training and greater collaboration amongst providers. Greater use might also be made of social media to communicate safety‐related concepts and the activation of community‐based services ought to be considered.

## CONFLICTS OF INTEREST

No conflicts of interest have been declared by the authors.

## AUTHOR CONTRIBUTION

AJ, ZCh: conceived and designed the study. AJ, ZCh, JB and MP: collected and independently coded the data. ZCh: was responsible for the drafting of the manuscript and made critical revisions to the paper for important intellectual content. AJ: gave final approval of the version to be published. All authors are accountable for accuracy or integrity of any part of the work.
