# Supplementary material for: Exploring the barriers to patient engagement in the delivery of safe care in Iranian hospitals: A qualitative study
Source: Nurs Open. 2019 Nov 25;7(1):457–65. doi: 10.1002/nop2.411 (PMC6917972; doi:10.1002/nop2.411)
Supplement: Supplementary file 1 [file NOP2-7-457-s001.docx]

**Supplementary Table- Statements made by participants during the interviews**

| **Themes** | **Subthemes** | **Illustrative statement** |
| --- | --- | --- |
| **Patient related barriers** | Low levels of health literacy | The level of health literacy amongst patients varies greatly… we have had patients that explained to us that they have different medications at home for blood pressure, high cholesterol and so on, but others don’t say anything.  Not all have a high level of health literacy, not everyone has medical or paramedical training. Few patients have a rich vocabulary; they easily speak inappropriately when they have questions or concerns…  …If we want to involve patients in patient safety, we have to prepare them by increasing their level of health literacy particularly about safety issues. |
|  | Ineffective patient education | The patient doesn’t need to know about all the processes, but they ought to know the minimum, … for example diabetics should know about how to take care of their feet and to manage their insulin.  Now patients know little about general health topics. But what about patient safety? What is a high-risk drug, how are they diagnosed or what blood test protocols does a hospital have? Patients know even less about these things.  Education is frequently patchy, it doesn’t take account of what information patients have and feedback isn’t provided… How have patients with an in-dwelling catheter been educated?  Education won’t be effective unless the doctor when connecting the patient’s serum, in a case of renal failure for example, educates the patient, without education the patient could change the infusion rate and the volume during the night because he doesn’t understand that these things matter…  …You can’t prescribe one approach to education for all patients. Sometimes it’s a priority for a patient to know about their medication and sometimes the priority is observing their pressure ulcer or repeating the referral test…  Although the reports contained details of the information that the patient had been provided with, none of this was true. Information that had not been provided has been recorded as provided. |
|  | Patient unwillingness | Sometimes side effects make a patient suspicious about their medication but they won’t say anything because they are frightened that if they do it will have a negative effect on their treatment. Because a patient is afraid of the consequences of expressing his dissatisfaction, he pretends to be satisfied.  …If the patient believes in the usefulness of the partnership, he will freely contribute. We need to show the positive impact that participation can have on the treatment and on safety.  Um… I remember when we had an infectious patient whose carer stayed with them, the nurse repeatedly told the carer that they must wash their hands when they touch the patient because if they didn’t they could get sick but the carer thought that it wouldn’t make any difference and took no notice of the nurse…  ... If, when we’ve told a patient not to leave the bed, and he does leave the bed, and nothing happens to him he’s not going to take any notice of anything the treatment team might say to him in the future… |
|  | Cultural barriers | Patients have different cultural backgrounds; different traditions and these may hinder their engagement in safer care.  Patients who have a different cultural background don’t cooperate with the diet plan, or continue to smoke despite the advice to stop, whose families bring particular types of food into the hospital. These patients feel that there isn’t a problem if they don’t listen to the advice given to them.  Sometimes it’s the doctors who provide little information that are considered to be good doctors and not the who provide detailed information. Sometimes there isn’t a culture of providing medical information. |
| **Staff related barriers** | Negative attitudes towards patient engagement | Having equipment that supports patient safety is not enough. It’s not enough just to have a wristband to identify the patient, to make sure the patient is properly identified the staff must also ask the patient for their name … Beliefs and attitudes also need to change.  If senior management doesn’t believe in and have a positive attitude towards engaging patients in patient safety, it’s impossible to do anything in the hospital.  In many cases, we have standards and even structures, for involving patients and their families in patient safety, but these are hindered by the reluctance of the providers who don’t believe in the involvement of volunteers and obstruct that involvement.  …Does the treatment team accept a patient's opinions? What’s the reaction when the patient asks; did you wash your hands? Some staff see this as interfering.  … Do staff have a positive attitude to complaints? Absolutely not! Staff see complaints as a something negative... At the moment, staff don’t have an acceptable attitude towards patient education and participation. They get annoyed. They think their time is being wasted and this annoys them.  …  So, it looks like they think it’s better to say nothing to the patient and, as a result we only ever see patients who agree with everything – the patient ends up being a “yes-man”  …a patient who is curious and who voices their concerns about errors is seen as being troublesome by the staff... |
|  | Lack of effective communication | Staff are on auto-pilot with the patients. Communication with the patient is extremely poor. Poor communication makes it difficult to implement measures addressing patient safety and patient involvement even when these measures might be approved of by nurses.  We don’t make patients feel comfortable, for example when we mark the surgical site ... there needs to be a friendly environment and easy communication... But even communication between staff is a major problem.  I think it's a very important that we listen to patents and not just talk to them. We should listen to them, what expectations do they have of us? Do they understand us? Get feedback from patients. Make them feel comfortable and at ease, tell us about side effects. This doesn’t happen often. We always talk and talk … but we don’t know if the patient has understood or not…  Doctors don’t even make eye contact when visiting patients. So, if there isn’t even any eye contact with a patient, how can the patients feel comfortable enough to ask questions or give feedback?  Despite the Ministry's emphasis on the use of the ISBAR^*^ model to ensure the exchange of relevant information and the avoidance of irrelevant information this model is rarely used correctly. |
|  | Workload | ...Wards are crowded, especially after the health reform plan. You see on a ward with 34 beds, all the beds are filled, and 8 extra beds have been set up in the hall and, in some outpatient departments beds have been set up in the waiting room for patients waiting to be transferred to the operating theatre. When you count there are 60 patients and with a workload as high as this I haven’t got the time to talk to the patients or to let them know what’s happening to them…  …the bed occupancy rate is 80… umm…not enough time, it’s impossible to increase patients’ awareness about, for example, reporting problems with medical devices.  For example, doctors who should be getting the patient’s informed consent or involving the patient in surgical checklists say that with 100 patients to look after they don’t have the time … it is difficult to convince them that involving the patient in a surgical checklist is necessary.  We (the providers) don’t accept patient participation in different aspects and in different places … The patient says he’s allergic to penicillin and the nurse (surprisingly) says: I’ll inject it slowly! And this leads to an adverse event. Nurses have a heavy workload but this by itself cannot be the cause of adverse events. If I just read the medicine label or listen to patient the right patient won’t have the wrong medicine...  Even though the staff might be very busy they need to be accountable…for example, a patient asking for help to avoid falling or who has a question about the time they want to take their tablet presses the nurse’s calling button, but nobody responds... that patient, and others, are left feeling distrustful. |
|  | Reluctance of physicians | Now, doctors don’t care … The head of the team of doctors should comply with the principles, and others in the team are expected to implement them. If they don’t, they can expect to be rebuked by a glance from the manager (head), he can’t say a word and does nothing. These are the contradictions actions speak louder than words.  … (as a doctor) I prefer not to explain DVT to the patient. I don’t explain that it’s a small clot which will lead to an embolism. I just say: don’t stay in bed, and I don’t explain why... without an explanation the patient won’t necessarily observe the instruction not to stay in bed.  Some doctors don’t wash their hands between patients... how can we expect a patient to ask the doctor to wash their hands when the doctor has shown no awareness of the need to do this?!… The impact of these people as role models for students during their training is greater than the impact of their classes …  Doctors think there’s no point in giving patients an explanation and are resistant to the idea that patients with lower levels of health literacy need to have an explanation … |
| **System related barriers** | Limited resources | Maybe the patient doesn’t like to share information about their background with the doctor in front of other people. Privacy doesn’t exist in many hospitals.  We have no facilities for the families of patients. They are displaced without even a place for them to sit. There are no facilities for informal caregivers whose money is taken from them to for the care of the patients.  The managers are trying to solve financial and human resource shortages every day. Pay the cost... Take the money from insurance companies... Financial challenges occupy managers’ minds and stop them thinking about other things like involving patients in safety. |
|  | The inadequate curriculum for health professionals | The college faculties provide a weak education plan. . Colleges don’t provide classes on the communication between the healthcare team and the patients. We just teach students how to deal with clinical symptoms, and we don’t educate them on how to deal with patients as people.  There is also a weakness in healthcare management as managers don’t learn clinical skills at university. A managerial perspective without any clinical understanding is a weakness. It causes mistrust between healthcare managers and others working in the healthcare system.  As far as I know, colleges offer is no educational content on how to get patients involved in patient safety. |
|  | Ineffective retraining programs | In many hospitals, patient safety experts are inexperienced, uninterested or not even trained… I myself didn’t know anything when I was appointed a patient safety coordinator, and I only learned by trial and error…  We didn’t have training programs that empower nurses to involve the patient. How to do it and how it’s necessary and helpful. We have a general training course for the staff, but often these courses go up to earn retraining points.  Many of the mistakes made by medical staff can be solved through training. Retraining programs can act as a tool to address the medical errors made by medical staff, for example, we assign many of the tasks that the nurse or particularly the assistant, were in charge of. Like changing the bedding and feeding the patient. These are the tasks for the medical staff, not patients. |
| **Community related barriers** | Poor disseminate information via the mass media | Using the mass media such as radio and television to provide information about, health and patient safety issues could empower patients.  TV channels don’t carry enough short animation clips and too few TV programs carry subtitles that support training.  General education is what’s required. Broadcasting is very helpful but also social media: WhatsApp &Telegram…  …their (patients) stay in hospital is short, and sometimes the training in hospitals is not sufficient. You know they are sick… umm... Some of the training given to patients needs to be repeated. A mechanism is needed to keep the training continuing through the use of mass media... |
|  | Lack of community based services | The WHO also mentions the use of the charitable groups and sites but these groups have a very low in our country.  The hospital becomes a place where patients, particularly those with a mental illness, remain for long periods and this increases the risks of getting hurt by other patients, which ultimately reduces levels of patient safety.  Sometimes the patient is missed and ends up staying in hospital for a long time s and the nurse thinks that not checking the blood pressure or controlling the patient's vital signs isn’t a problem.  It is better that community-based organizations provide general training and patients are empowered before they come into hospital , because once in hospital the patient is only thinking about his illness and is not very interested in having training.  The other thing is that I think, uhh… the readmission of patients after discharge, this is why post discharge care is very important and this can be done through community-based clinics…  The law emphasizes a TOP-DOWN approach to the provision of health services. This approach does not allow for participation. |

^*^ Identify Situation, Background, Assessment, Recommendation
